# ICD Pocket-Site Infection Secondary to Gonococcal Bacteremia: The First Reported Case

**DOI:** 10.1155/2021/9250967

**Published:** 2021-05-04

**Authors:** Sardar Hassan Ijaz, Ali Haider Jafry, Areeba Shahnawaz, Mark Allee

**Affiliations:** ^1^Department of Cardiovascular Disease, Lahey Medical Center, 41 Mall Road, Burlington, MA 01805, USA; ^2^800 Stanton L. Young Blvd, AAT 6300, Department of Medicine, University of Oklahoma Health Sciences Center, Oklahoma, OK 73105, USA; ^3^Faisalabad Medical University, Sargodha Road, Faisalabad, Punjab 38000, Pakistan

## Abstract

**Introduction:**

Cardiovascular implantable electronic devices (CIEDs) are being increasingly used in the primary and secondary prevention of malignant ventricular arrhythmias and conduction system disorders. Infectious complications associated with CIEDs include infective endocarditis, lead infections, and pocket-site infections, primarily involving *Staphylococcus* species. Infective endocarditis is a rare but life-threatening complication of gonococcal bacteremia. We report the first case of a CIED pocket-site infection secondary to *Neisseria gonorrhoeae (N. gonorrhoeae)*.

**Case:**

A 56-year-old male with a history of congestive heart failure status postimplantable cardioverter-defibrillator (ICD) insertion presented with a pocket-site swelling initially concerning for a hematoma which began to exhibit erythema and tenderness. The patient reported a history of high-risk sexual behavior. On presentation, he was afebrile and hemodynamically stable. Physical exam showed a 5 cm × 6 cm pocket-site swelling with overlying erythema. Labs revealed elevated ESR and CRP levels. Transthoracic and transesophageal echocardiography was concerning for infective endocarditis and lead vegetations. Blood cultures tested positive for *N. gonorrhoeae*. He underwent surgical debridement with complete ICD extraction and drainage of infected serosanguineous pocket fluid. Tissue cultures were negative, but isolation of *N. gonorrhoeae* in blood cultures confirmed it as the causative agent of the pocket-site infection in the absence of prior Gram-positive coverage. He was started on a prolonged course of ceftriaxone for 4 weeks with reimplantation of ICD at a different site after completion of treatment.

**Conclusion:**

In patients with high-risk sexual behavior, gonococcal bacteremia can potentially lead to CIED infection. These individuals should be prudently evaluated for infective endocarditis or pocket-site infections as presenting complaints can be subtle.

## 1. Introduction

The use of cardiovascular implantable electronic devices (CIEDs) has increased significantly for the prevention of life-threatening arrhythmias in coronary artery disease and congestive heart failure [1]. CIEDs comprise implantable cardioverter-defibrillators (ICDs), pacemakers, and cardiac resynchronization therapy devices. Concordant with this trend is a rising incidence of CIED-related infections, with ICDs contributing to a lifetime risk of 1.9% [2–4]. A number of host, procedure, and device-related risk factors have been studied, with recent device manipulation or exchange being the most significant [5]. *Staphylococcus* species (*Staphylococcus aureus* and coagulase-negative *Staphylococci*) have been the usual culprit in infectious complications [6]. We discuss the first reported case of a pocket-site and lead infection secondary to *N. gonorrhoeae* bacteremia in an individual with a history of high-risk sexual behavior.

## 2. Case Presentation

A 56-year-old African-American male with past medical history of paroxysmal atrial fibrillation (on apixaban), chronic obstructive pulmonary disease (COPD), chronic kidney disease (CKD) stage III, and heart failure with reduced ejection fraction (EF: 25–30%) status post-ICD placement for primary prevention presented to the arrhythmia clinic with a pocket-site swelling. The patient reported that the swelling began recently a few days after lifting a heavy object at home. He had previously undergone complete ICD device and lead extraction and subsequent reimplantation 2 years prior due to a pocket-site infection which was secondary to methicillin-resistant *Staphylococcus aureus* (MRSA). Pocket-site ultrasound in clinic revealed a 4.5 cm × 2 cm × 2 cm fluid collection concerning for hematoma, prompting discontinuation of apixaban. The swelling progressively increased in size on a subsequent clinic visit. This, along with the development of pocket-site pain, erythema, and tenderness, in association with generalized malaise and fatigue prompted admission.

On presentation, he was afebrile (98.3°F) and hemodynamically stable (pulse 93 bpm and blood pressure 115/82 mm Hg) with oxygen saturations of 96% on room air. Physical examination revealed a 5 cm × 6 cm precordial swelling exhibiting localized warmth and redness. A new-onset II/VI systolic murmur was auscultated in the tricuspid area. Relevant laboratory workup showed no leukocytosis (WBC 8,100/mm^3^) but elevated erythrocyte sedimentation rate: 34 mm/hr; C-reactive protein: 70.9 mg/dL; procalcitonin: 1.31 ng/ml; and INR: 1.3. Transthoracic echocardiography showed reduced EF (25–30%) with small mobile echodensities associated with the lead in the right ventricle which were concerning for vegetations. Transesophageal echocardiography showed no definitive ICD lead vegetations; however, the right atrium had a filamentous structure suspicious for an early infected vegetation. Meanwhile, blood cultures grew Gram-negative cocci in ¼ bottles. Since the patient had reported a prior penicillin allergy, aztreonam was initiated. The Gram-negative cocci speciated to *N. gonorrhoeae* on blood cultures, with the patient divulging a history of high-risk sexual behavior (unprotected, multiple sexual partners) in the recent past. Of note, he did not report any urogenital symptoms during the course of his illness. Further clarification of his penicillin allergy, which was limited to hives, allowed for transition to intravenous (IV) ceftriaxone on the second day of antibiotic therapy. Repeat blood cultures 24 hours later also grew gonococci but cleared thereafter. The patient underwent surgical debridement and complete device and lead extraction a week later which revealed infected and necrotic tissue at the pocket-site with drainage of serosanguineous fluid. The pocket site was washed with a triple-antibiotic solution and closed. Cultures from the tissue, fluid drained from the pocket, and the device failed to grow any organisms while Gram stain was negative for any pathogens as well. Infectious disease (ID) was consulted. Given the isolation of *N. gonorrhoeae* alone on blood cultures and clinical evidence of pocket-site infection, a diagnosis of device and pocket-site infection secondary to gonococcal bacteremia was made. In the absence of prior exposure to antibiotics with Gram-positive coverage, pocket-site infection with Gram-positive organisms, including the commonly implicated *Staphylococcus* or *Streptococcus* species, was effectively ruled out as the etiology of the infection. In accordance with ID recommendations, he continued to receive IV ceftriaxone for 4 weeks with gradual resolution of the pocket-site swelling. Since the ICD was implanted for primary prevention of ventricular arrhythmias, it was deemed prudent to wait until the patient had completed an appropriate course of antibiotics. He responded well to this course of therapy and was successfully discharged. He continued to follow with ID and cardiology as outpatient, eventually undergoing successful reimplantation of an ICD 1 month later at a different site without complications.

## 3. Discussion

CIED infections encompass pocket-site and systemic (infective endocarditis or lead infection) subtypes. These subtypes are not mutually exclusive, as demonstrated in our patient who exhibited both a pocket-site infection and infective endocarditis (IE). Infections may also be classified as primary or secondary; our case exhibited CIED infection secondary to gonococcal bacteremia acquired as a sexually transmitted disease. *S. aureus* and coagulase-negative *Staphylococci* contribute to a majority (70–90%) of cases, with Gram-negative bacilli and fungal pathogens causing the remainder [6, 7]. Cultures from pocket site, lead tips, and blood samples may be, respectively, positive only in 61%, 79%, and 77% of all patients according to one study [8]. In addition to a previous episode of device revision/replacement and pocket reoperation, our patient also exhibited some other significant risk factors for infection. These risk factors included CKD, COPD, congestive heart failure, and anticoagulant use [8, 9].

Endocarditis may be reported in 1-2% of patients with disseminated gonococcal infection, making this a relatively rare entity with only a handful of cases reported in the medical literature [10, 11]. Symptoms may be subtle, requiring a high index of suspicion in patients, particularly those with CIEDs. If a diagnosis of pocket-site infection is suspected on physical examination indicating local inflammation, echocardiography and blood cultures should be performed to evaluate for lead infection and IE. No cases have been reported in the literature describing gonococcal pocket-site infections.

Corroborating prior reports, gonococcal growth on tissue and device cultures was negative in our case, likely due to prior antibiotic administration for a week, with the fastidious nature of the organism contributing to it as well. *N. gonorrhoeae* endocarditis leads to significant valvular damage, necessitating surgical intervention in up to 70% of cases, compared to 25–50% when other organisms are involved [10].

CIED infections are managed with IV antibiotics, device explantation, debridement, and reimplantation at a different site [7]. A prolonged course of antibiotics after device revision is employed depending on the etiology of the infection, with a minimum of 4 weeks reserved for IE or persistent bloodstream infection (BSI) despite device removal [12]. Device reimplantation is delayed in cases of persistent BSI until resolution of bacteremia, with successful replacements being reported as early as 3 days or up to 2 weeks after resolution of bacteremia. Recurrent infections can be prevented by the use of prophylactic antibiotics, antibiotic pouches, or use of subcutaneous ICDs.

## 4. Conclusion

Gonococcal bacteremia can lead to CIED pocket-site infection necessitating device removal, surgical debridement, and systemic antibiotics. Device infections and endocarditis with *N. gonorrhoeae* should be part of the differential diagnoses in patients with high-risk sexual behavior presenting with constitutional symptoms and/or new-onset heart murmurs.

## Data Availability

The data that support the findings of this study are available from the corresponding author upon reasonable request.
